# Pathologic and Radiologic Correlation of Adult Cystic Lung Disease: A Comprehensive Review

**DOI:** 10.1155/2017/3502438

**Published:** 2017-02-08

**Authors:** Prajwal Boddu, Vamsi Parimi, Michale Taddonio, Joshua Robert Kane, Anjana Yeldandi

**Affiliations:** ^1^Department of Internal Medicine, Advocate Illinois Masonic Medical Center, 836 West Wellington Avenue, Chicago, IL 60657, USA; ^2^Department of Pathology, Loyola University Medical Center, 2160 S. 1st Avenue, Maywood, IL 60153, USA; ^3^Department of Radiology, UC San Diego, 200 West Arbor Drive, San Diego, CA 92103, USA; ^4^Department of Anatomic Pathology, Feinberg School of Medicine, Northwestern University, 303 E. Chicago Ave., Chicago, IL 60611, USA; ^5^Northwestern University, Chicago, IL, USA

## Abstract

The presence of pulmonary parenchymal cysts on computed tomography (CT) imaging presents a significant diagnostic challenge. The diverse range of possible etiologies can usually be differentiated based on the clinical setting and radiologic features. In fact, the advent of high-resolution CT has facilitated making a diagnosis solely on analysis of CT image patterns, thus averting the need for a biopsy. While it is possible to make a fairly specific diagnosis during early stages of disease evolution by its characteristic radiological presentation, distinct features may progress to temporally converge into relatively nonspecific radiologic presentations sometimes necessitating histological examination to make a diagnosis. The aim of this review study is to provide both the pathologist and the radiologist with an overview of the diseases most commonly associated with cystic lung lesions primarily in adults by illustration and description of pathologic and radiologic features of each entity. Brief descriptions and characteristic radiologic features of the various disease entities are included and illustrative examples are provided for the common majority of them. In this article, we also classify pulmonary cystic disease with an emphasis on the pathophysiology behind cyst formation in an attempt to elucidate the characteristics of similar cystic appearances seen in various disease entities.

## 1. Introduction

 Cystic air spaces represent sustained, unresolved insults to the pulmonary airways and parenchyma. Reversibility of these cysts depends on the stage in the natural history of a particular disease entity and on their response to resolution of the inciting insult [1]. Radiological classifications of lung cysts have suffered from standardization due to a lack of a uniform definition. Nomenclature has undergone periodic revisions, and the most recent glossary of definitions has been based on the radiological anatomy descriptions, as recommended by the third Fleischer Society committee meeting. These definitions have helped categorize cystic lung disease broadly into “true cystic,” “cavitary,” and “cystic mimics.” For the purpose of this discussion, we will include and study cavitary lung disease and cystic disease mimickers under the broad umbrella of cystic lung disease.

As per the third Fleischer Society committee meeting criteria, a cyst may be defined as any low-attenuating circumscribed space, containing gas or liquid that is enclosed by an epithelial or fibrous wall and has a well-defined interface with the normal lung tissue [2]. A cavity, on the other hand, is a gas-filled space within a pulmonary consolidation or mass that is characterized by markedly thicker walls [2]. A lung cyst is usually less than 4 mm in wall thickness. By contrast, cavities have walls greater than 4 mm in thickness [3]. Diseases characterized by true cysts are relatively uncommon and are usually distinguishable from their mimicking entities like bullae, honeycombing, and bronchiectasis [4]. Bullae are sharply demarcated areas of emphysema with a wall thickness of less than 1 millimeter [2, 3, 5]. Pneumatoceles are thin-walled, gas-filled spaces occurring in association with acute infections and after trauma [2, 3]. Bullae can grow to larger volumes and occupy up to an entire lobe, while pneumatoceles tend to resolve with the improvement of the underlying infection [6]. Bullae and pneumatoceles are considered subcategories of cysts [2, 3]. Other important mimics include honeycombing, bronchiectasis, and loculated parapneumonic effusions [3, 7, 8]. Honeycombing refers to a patterning of irregular, thick-walled air spaces [2]. This condition is a sign of end stage pulmonary fibrosis. Although several disease processes present with similar pathologic and radiologic features, honeycombing is considered specific to usual interstitial pneumonia (UIP) [6, 9]. Honeycombing on CT allows distinction of UIP from other idiopathic interstitial pneumonias [7]. On the other hand, emphysema is characterized by abnormal air spaces distal to the terminal bronchiole with destruction of the alveolar walls. Emphysematous bullae may be differentiated from cysts on CT by their indefinable walls and decreased vascularity [3]. Bronchiectatic air spaces, when viewed “en face,” may carry a similar appearance to cysts creating significant diagnostic confusion. However, the presence of bronchovascular bundles adjacent to air spaces can help differentiate bronchiectasis from true cysts.

One of the most important factors of cyst causation is the presence of interstitial abnormality upstream to the site of cyst formation, creating a ball valve effect. These check valves prevent egress of air on exhalation while allowing air to enter on inspiration. The coexistence of interstitial abnormalities in a small airway disease may be required for cyst formation. This supposition is supported by the lack of cystic air spaces as a pathologic or HRCT finding in pure bronchiolitic disorders without peribronchiolar infiltration [4]. Other mechanisms of cyst formation include traction bronchiectasis/bronchioloectasis, alveolar wall dissolution and conflation, and local emphysema from collateral air drifts into collapsed acini distal to obstructed bronchioles [1, 10].

## 2. Cystic Lung Disease

We classified cystic lung diseases based on their dominant mechanism of cyst formation (as in Table 1). This mechanistic classification is an attempt to offer better insights into the association between histopathology and radiologic characteristics. It is important to keep in mind that such mechanisms are neither exhaustive nor mutually exclusive in causation and more than one mechanism may contribute at any specific phase to disease evolution. It must also be noted, from Table 1, that some of the disease entities (e.g., lung micrometastasis) may present with atypical radiologic manifestations consequent to cystic mechanisms less frequently operating in that disease entity. These atypical presentations will be discussed further under individual disease entities.

We have subdivided cystic lung disease entities based on underlying cystic mechanism into (1) cystic dilation of lung structures: (a) ball valve effects, (b) traction bronchiectasis/alveolar ectasia, and (c) cystic suppurative/necrotic bronchiectasis; (2) parenchymal necrosis: (a) suppurative, (b) caseous, (c) ischemic, and (d) intratumoral; (3) alveolar rupture and air space conflation; (4) cyst expansion with lung displacement: (a) infectious, (b) congenital, and (5) miscellaneous.

### 2.1. Cystic Dilation of Lung Structures

#### 2.1.1. Ball Valve Effects

**Figure 1:**
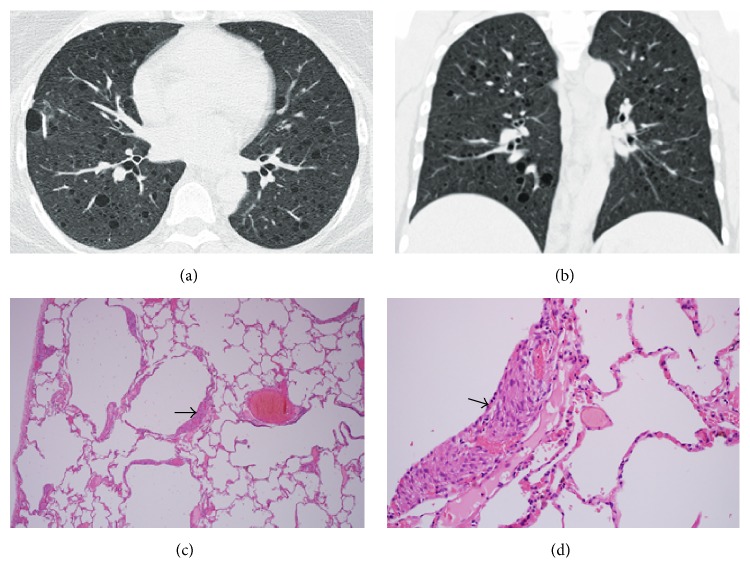
Lymphangioleiomyomatosis (LAM). High-resolution CT scan in axial (a) and coronal (b) views demonstrating numerous well-defined, thin-walled lung cysts varying in size from 2 to 18 mm. There is diffuse involvement of all lung fields, and the lung parenchyma between the cysts is normal. (c) Thin-walled cystic air spaces interspersed between uninvolved lung parenchymata (magnification ×40). (d) Focal cyst wall thickening from smooth muscle proliferation (arrow) (magnification ×100).

**Figure 2:**
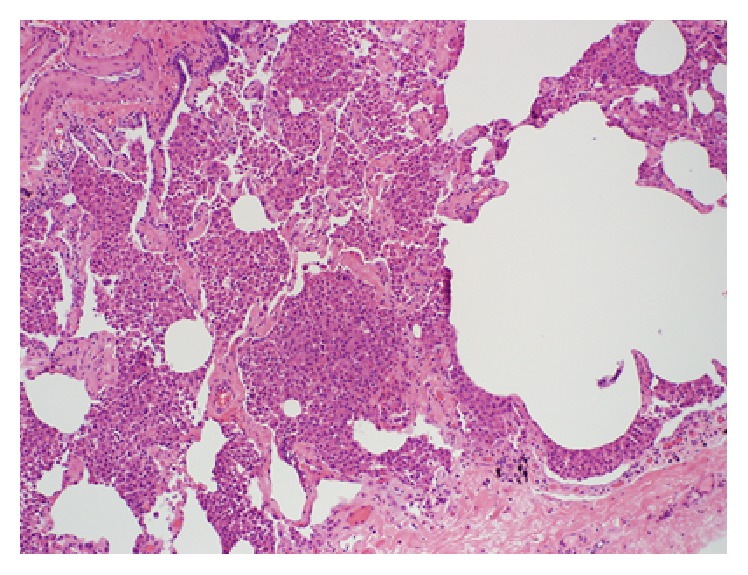
Histopathology image of a RB-ILD specimen demonstrates diffuse accumulation of macrophages in the peribronchiolar, bronchiolar regions. Interspersed among the macrophage collections are alveolar septae with a large cystic air space on the right (magnification ×100).

*(i) Lymphangioleiomyomatosis (LAM)*. LAM is an uncommon interstitial lung disease that affects women in the reproductive age group [3, 11, 12]. It may occur sporadically or in association with tuberous sclerosis caused by mutations in one of the two genes, TSC1 and TSC2 [3]. The clinicoradiologic picture is characterized by progressive pulmonary cystic changes and chylous pleural effusions [12]. Morphology is characterized by the presence of nodules, composed of two dimorphic populations of abnormally proliferating cells [3]. Proliferation of smooth muscle cells along the peribronchiolar lymphatics leads to bronchiolar obstruction and formation of lung cysts [11] (see Figure 1). Proliferation of smooth muscle cells of the lymphovascular structures results in blocking of the axial lymphatics by lymphangiomyoma which leads to chylous effusions and ascites. Centrilobular nodules on the CT correspond to type 2 pneumocyte hyperplasia. The smooth muscle proliferation represents focal ground-glass opacities; the lymphatic obstruction is seen as septal thickening [3, 11]. The characteristic CT findings in LAM are randomly distributed, diffuse thin-walled cysts, ranging from 0.5 to 2 cm in diameter, surrounded by normal lung parenchyma [12] (see Figure 2). The cyst size may vary proportionately with the severity of the disease. These LAM cells have partial melanocytic differentiation and stain positive for HMB-45 (a glycoprotein occurring in the premelanosomes in the cytoplasm). In contrast to PLCH, cysts in LAM are evenly distributed throughout the parenchyma and are more regular in shape and more uniform in size [11]. Nodules are characteristically absent in LAM and, if rarely present, are not larger than 10 millimeters in size [13, 14]. A combination of random cyst distribution and absence of ground-glass distribution helps distinguish LAM from other disease entities. In fact, the diagnosis can be arrived at with a reasonably high degree of accuracy without requiring a biopsy [14].

*(ii) Respiratory Bronchiolitis-Interstitial Lung Disease (RB-ILD) and Desquamative Interstitial Pneumonia (DIP)*. Interstitial lung disorders (ILD) are a heterogeneous group of entities with similar clinical, radiologic, physiologic, and pathologic features. Pulmonary Langerhans cell histiocytosis (PLCH), RB-ILD, and DIP are a group of smoking related interstitial lung disorders characterized by cysts. RB-ILD and DIP share clinical and histological similarities and represent different manifestations of the same disease continuum. RB-ILD/DIP is characterized by accumulation by pigmented macrophages around the terminal airways with mild interstitial inflammatory changes which are confined to peribronchiolar parenchyma in case of RB-ILD and diffusely involve parenchyma in case of DIP [7]. RB-ILD predominantly involves the peripheral lower lobe and is typified by the occasional presence of centrilobular nodules and areas of patchy ground-glass opacities (due to alveolar inflammation and accumulation of intra-alveolar macrophages) [14, 15]. DIP is a more diffuse parenchymal reaction and is characterized by basally predominant ground-glass opacities (corresponding to intra-alveolar macrophages and minimal fibrosis) (Figure 2) [7]. Cysts are not a predominant feature of either disease and usually arise late in presentation as result of the ball valve effect from bronchiolar stenosis. On the other hand, cystic changes seen are generally emphysematous areas as a direct result of smoking. These hardly perceptible, thin-walled cysts arising in areas of ground-glass attenuation are features that characterize RB-ILD/DIP and they help differentiate this entity from the pathophysiologically related emphysema, where cysts are not surrounded by ground-glass attenuation [14]. The cysts are reversible in nature and may resolve with time unlike in the case of UIP [16].

*(iii) Pulmonary Lymphoproliferative Disorders*. Lymphoproliferative disorders comprise a range of disease entities and are classified into reactive or neoplastic on the basis of cellular morphology and presence of clonality [17].

**Figure 3:**
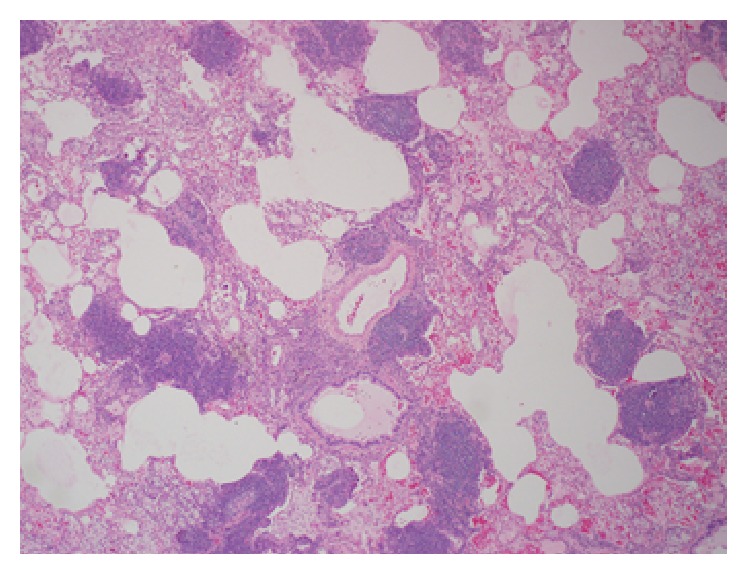
Pathology of lung biopsy specimen from a patient with lymphoid interstitial pneumonia. (a) Low power view showing peribronchiolar and interstitial lymphocytic follicular aggregates alongside dilated cystic air spaces (magnification ×40). (b) Low power view of another LIP specimen with diffuse lymphocytic follicular aggregates infiltrating the interstitium (magnification ×40).

*Lymphocytic Interstitial Pneumonia (LIP)*. Benign reactive pulmonary lymphoproliferative diseases comprise three disease entities: follicular bronchiolitis, nodular lymphoid hyperplasia, and LIP [18]. LIP can be idiopathic or can occur in association with autoimmune disorders (Sjögren's syndrome), infections (*P. jiroveci*, HIV, hepatitis B, and Epstein-Barr virus), collagen vascular diseases, and immunodeficiency states (AIDS, SCID), among others [3, 11, 19, 20]. LIP is characterized microscopically by a polymorphous inflammatory infiltrate of lymphocytes, plasma cells, and histiocytes that diffusely expand the alveolar septa (Figure 3) [3, 20]. Lymphocyte predominant areas involving the bronchovascular bundles, interlobular septae, and subpleural areas are affected. The characteristic HRCT findings consist of small-sized centrilobular nodules (<10 mm), ground-glass opacities, and scattered, randomly distributed, thin-walled cysts [14, 20]. Cysts are seen in more than half the cases of LIP and are generally distributed randomly without clustering [21]. GGOs are due to the diffuse interstitial inflammation while centrilobular branching nodules are due to peribronchiolar cellular infiltration. The cysts are formed due to partial bronchial obstruction due to peribronchiolar infiltration [19]. The cysts are reversible on resolution of disease. They are typically few in number, measuring less than 3 cm in diameter. The presence of cysts and the absence of consolidation and effusion rule in favor of LIP when compared to a lymphoma [17]. In contrast to the perilymphatic distribution of LIP, follicular bronchiolitis involvement is limited to the airways with minimal peribronchial disease and lack of cysts on CT [11, 17]. CT findings are frequented by interlobular septal and bronchovascular thickening, which in combination with random cystic distributions help distinguish and differentiate LIP from other disease entities.

*Lymphoma*. Malignant lymphoproliferative disease may be primary or secondary in nature. The most common primary pulmonary malignant lymphoproliferative disorder is MALToma [17]. MALT lymphoma can present similarly to LIP histopathologically and radiologically, posing diagnostic difficulty. However, the presence of cysts is far more common in LIP and helps in its differentiation [17]. Most importantly, it is imperative to recognize and differentiate lymphoma from LIP as the treatment and prognosis are markedly different. Honda et al. [22] reported characteristic findings on CT which help differentiate LIP from lymphoma, cysts being characteristic of LIP and presence of consolidations with large nodules and pleural effusions being characteristic of lymphoma. It must be kept in mind that radiologic findings vary depending on the histological subtype of lymphoma. More aggressive high grade lymphomas occurring in immunosuppressed patients have more nodular presentations [23]. It is worthwhile to note that lymphomas can also cavitate and present with similar radiologic findings as solitary bronchogenic carcinomas [3, 17].

Secondary pulmonary lymphomas, including Hodgkin's and non-Hodgkin's lymphomas, can less frequently involve the lung parenchyma with varied and nonspecific imaging features, ranging from solitary nodules to parenchymal lymphangitic involvement [17]. A study by Filly et al. [24] analyzing the incidence of intraparenchymal involvement in HL and NHL revealed nodules and cysts in only 11.6% of Hodgkin's disease patients and an even lower percent in the NHL population.

Sakashita et al. [25] described a case of acute T-cell lymphoma in which there was an alloy wheel appearance with a large cyst in the center of the ground-glass opacities surrounded peripherally by smaller clustered cysts from a combination of check valve effect and traction bronchiolectasis.

**Figure 4:**
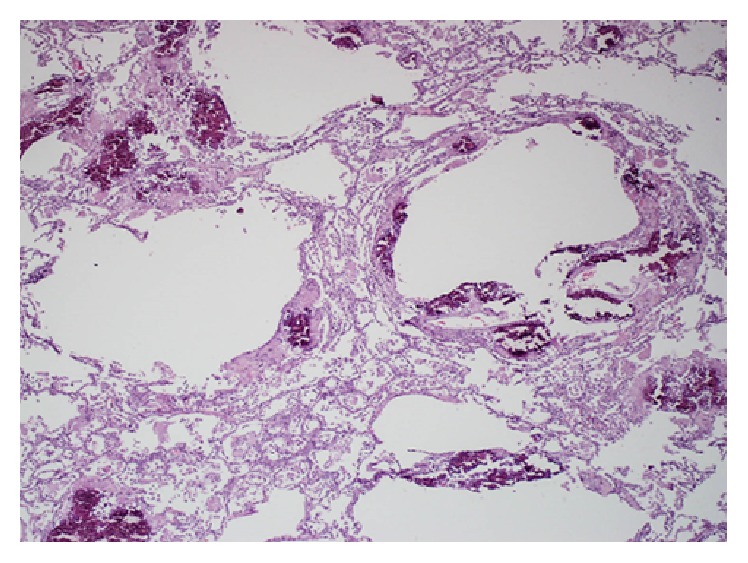
A patient with* Pneumocystis jirovecii* pneumonia. Low power view showing thin-walled cystic air spaces lined by inflammatory cells and necrotic debris with dystrophic calcification; inflammatory exudate in the interstitium and alveolar septae (magnification ×40).

*(iv) Pneumocystis jiroveci Associated Multiple Cystic Lung Disease*.* Pneumocystis jiroveci* has emerged as an important opportunistic pathogen in immunocompromised patients. The typical pulmonary features of* P. jiroveci* infection consist of diffuse pulmonary ground-glass and reticular opacities usually without pleural effusion and lymphadenopathy. However, the radiological features of* P. jiroveci* pneumonia have changed since the epidemic of AIDS from a classic CT pattern of extensive ground-glass attenuation [6] to lung cysts with an upper lobe distribution of parenchymal opacities. The formation of cystic lesions is attributed to long-standing low grade inflammation before a clinical diagnosis of PCP is made [26]. The latter radiological picture has become less common, due to early intervention with antiretroviral and PJP prophylaxis therapy [27]. Pathologically, the extensive ground-glass opacities in PJP correspond to accumulation of intra-alveolar fibrin, necrotic debris, and organisms. In patients without HIV infection, the extent of ground-glass opacity tends to be greater, reflecting pulmonary damage by an active host inflammatory immune response. The cystic lesions (Figure 4) disappear or reduce in size following treatment (as in pneumatoceles of another infectious origin). The presence of extensive GGOs with apically predominant cysts is indicative of PCP in AIDS patients. Of note, PCP lesions may infrequently manifest as cavitary infiltrates making a diagnosis extremely difficult [28].

#### 2.1.2. Traction Bronchiolectasis/Alveolar Ectasia

*(i) Interstitial Lung Disorders*

**Figure 5:**
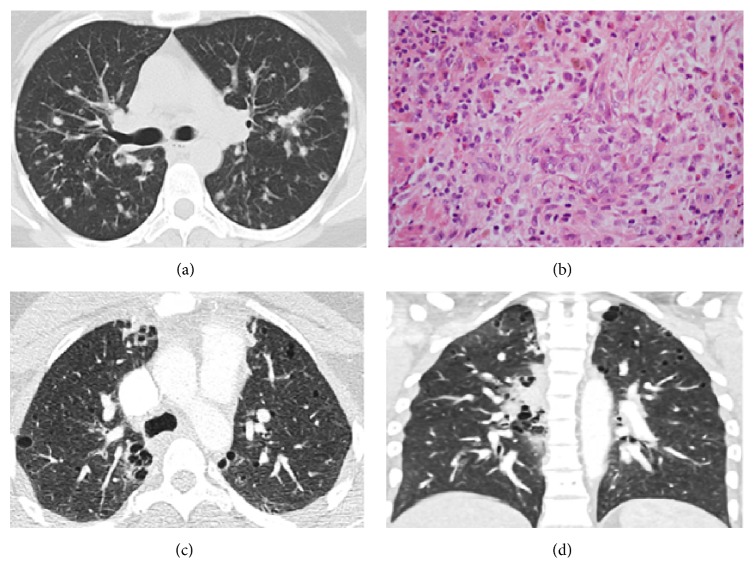
Pulmonary Langerhans cell histiocytosis. HRCT axial view (a) demonstrating a nodular phase of PLCH with multiple bronchiolocentric variable-sized nodules. The nodules correspond to granulomas on histology (b) showing high power view of Langerhans histiocytic cell aggregates surrounded by lymphocytes, eosinophils, and plasma cells (magnification ×400). Nodular phase eventually evolves to a cystic phase. Axial (c) and coronal (d) views demonstrating multiple subcentimeter lung cysts with thickened and irregular walls. Coronal view shows predominance of these lesions in the upper lobes, with relative sparing of the lung bases.

*Pulmonary Langerhans Cell Histiocytosis (PLCH)*. Pulmonary Langerhans cell histiocytosis (PLCH) is an isolated form of LCH involving the lung. The most characteristic radiological presentation is the presence of apical nodules and cysts in young adult smokers [29]. Morphology is characterized by peribronchiolar Langerhans cell infiltrates forming stellate nodules (see Figure 5(b)). The nodular lesions may cavitate to form thick-walled cysts [11]. Some of these cystic nodules enlarge with increasing degrees of cicatrization, resulting in irregular thin- or thick-walled cysts [14] (see Figures 5(a), 5(c), and 5(d)). The cellular infiltrates disappear, leaving behind fibrotic scars surrounded by distorted and enlarged air spaces, typically 2 mm to 2 cm in diameter [29]. Bronchiolocentric cellular nodules correspond to the radiographic nodules on the CT and the fibrous cavitations correspond to the cysts [3]. The cysts enlarge and coalesce to form bizarre shaped cystic air spaces [3, 30]. Cystic air spaces in PLCH may be distinguished from the round cysts in LAM and LIP by their distorted shapes as well as by their characteristic apical location [29]. Cyst configurations in PLCH are more frequently lobulated than in other diseases. Centrilobular emphysema may be distinguished from PLCH by the lack of a perceptible wall and the central location of vascular structures in the former [6, 11]. PLCH shares it cystic pathogenesis with usual interstitial pneumonia which is distinguished by clustered distribution of its cysts preponderant in the lower lung zones [14].

#### 2.1.3. Cystic Suppurative/Necrotic Bronchiectatic Air Spaces

**Figure 6:**
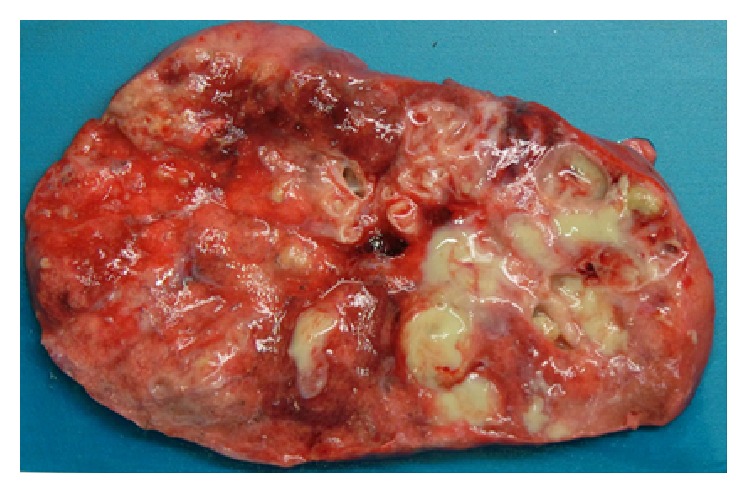
Gross specimen of resected lung from a patient with cystic fibrosis. Extensive cystic bronchiectasis with pus filled bronchial air spaces, dilated bronchi extending into the periphery.

*(i) Bronchiectasis*. Bronchiectasis is irreversible localized or diffuse bronchial dilatation, usually resulting from chronic infection, proximal airway obstruction, or congenital bronchial abnormalities [1]. HRCT criteria include the signet ring sign, lack of normal tapering of the bronchi towards the periphery such that bronchi are seen within a centimeter of the pleural surface [9, 31]. The signet ring appearance corresponds to a cross section of dilated bronchus adjacent to its accompanying pulmonary artery [9]. Tree-in-bud pattern and grape-like clusters in the peripheral airways are other signs of bronchiectasis seen on CT. The tree-in-bud appearance is due to mucous debris within the airways and the grape-like clusters are due to the resultant air trapping [6, 31]. Bronchial wall thickening, despite being frequent, is not necessarily diagnostic with thin-walled spaces occurring in congenital forms of bronchiectasis [32]. Bronchiectasis is caused by a vicious cycle of inflammation and an altered response to infection [33]. Normal mucosal and muscular layers fail to heal because of repeated infectious insults. Transmural inflammation occurs, not only destroying important supportive mural tissues such as smooth muscle and cartilage causing bronchomalacia, but also resulting in scarring of the wall, leading to both airway thickening and dilatation of the airways up to the periphery of the lung [34] (Figure 6). Bronchomalacia can make bronchi excessively collapsible and further air trapping [35]. CT is the gold standard for diagnosis and grading of disease severity in bronchiectasis. Disease severity is graded on the basis of the severity of bronchial dilatation, as cylindrical or tubular, a mild form; varicoid, a moderate form; and cystic or saccular, a severe form [9, 31]. Bronchiectasis can be distinguished from pulmonary cysts by following the dilated airways on multiple sequential CT slices [36]. Cystic bronchiectasis may be differentiated from bullae by the difference in the inspiration and expiration CT. Cystic bronchiectatic spaces communicate with the airways and show an increase on the inspiration CT and reduction on expiration CT whereas bullae remain largely unchanged regardless of the phase of respiration [36]. The underlying causes of bronchiectasis, for example, due to infectious or fibrosing disease, may be diagnosed in the appropriate clinical context by their zonal cystic distributions and ancillary pulmonary radiologic findings [37]. Another disorder associated with cavities is bronchiolitis obliterans organizing pneumonia, an organizing pneumonic process triggered by a variety of insults including drugs, toxins, autoimmune disease, and viral pathogens [38]. Cavitations occur in a small minority of cases and are nonspecific, thus warranting a lung biopsy for diagnosis [39].

### 2.2. Parenchymal Necrosis

#### 2.2.1. Suppurative

Infectious cavities may occur during or subsequent to a necrotizing pneumonic process* (Staphylococcus aureus or Streptococcus pneumonia)*, postprimary tuberculosis (upper lobe preference) [3, 30, 40], or chronic fungal infections like* Mucor* [3] (Figure 12). Pulmonary actinomycosis, which can mimic a bronchogenic carcinoma [30], may present as a persistent pulmonary mass or consolidation with cavitations [41]. Atypical causes of cavities that are not frequently seen outside endemic areas include paragonimiasis and echinococcosis and should be considered depending on the clinical setting [3, 42, 43]. Unlike in purely cystic lung disease, pathogenic causes of cavitary lesions are not easily predicted by radiographic algorithms and require supplementary microbiological and pathological evaluations [44].

*(i) Lung Abscess*. A lung abscess is defined as a lesion formed by pulmonary tissue necrosis with cavity formation from expulsion of necrotic debris. Lung abscesses are recognized by their round or oval configuration, presence of air-fluid levels, and thick walls. Pneumonia complicated by the formation of multiple, small (less than 2 cm) abscesses is referred to as necrotizing pneumonia. Abscesses may be classified as either primary or secondary depending on the presence of a preexisting condition. A primary abscess is caused by infection in an otherwise healthy host. A secondary abscess may be secondary to postobstructive pneumonia, bronchiectasis (Figure 10), or extrapulmonary or hematogenous spread of infection, among others. Aspiration is the most common cause of pulmonary abscesses, making the superior segment of the right lower lobe the most common site for infection. CT is the most sensitive and specific imaging modality to diagnose a lung abscess. Acute abscesses, over time, develop a chronic inflammatory layer of lymphocytes, plasma cells, histiocytes, and fibrosis around the pyogenic membrane creating better defined margins [45]. The typical appearance is that of a cavity containing an air-fluid level corresponding to necrotic debris and infected fluid, surrounded by a thick wall corresponding to a layer of fibrosis formed from chronic inflammation. CT distinction may be difficult between true abscesses and their radiologic mimickers, including cavitary neoplasms (see below). This may be resolved with the administration of contrast which helps identify abscess margins from the surrounding consolidation. Cavities are a very rare feature of emboli/infarcts and their presence in a case of suspected pulmonary infarct indicates septic embolization [5].

*(ii) Pneumatocele*. Pneumatoceles are usually transient cysts resulting from an inflammatory process that causes central necrosis with subsequent retraction by surrounding pulmonary tissue. The mechanism is primarily a combination of parenchymal necrosis and check valve obstruction [9]. The most common cause is infectious, often in patients with a localized bacterial pneumonia. Pneumatoceles differ in appearance from abscesses by way of their air-filled cavities and thin, regular walls. The cyst is frequently larger than the preceding cavitation due to resulting expansion from elastic recoil by surrounding lung. Infectious pneumatoceles are most commonly seen in staphylococcal and streptococcal infections but have also been seen in pneumococcus and Gram negative infections [3, 5, 46]. Pneumatoceles may also be seen in immunocompromised patients due to* Pneumocystis jiroveci* infection (see above). Infectious cysts that persist despite resolution of primary infection should raise suspicion of less common infections like* Pneumocystis* or* Echinococcus*. Pneumatoceles may also occur as a complication of blunt trauma [47, 48] or aspiration of hydrocarbon fluid [5, 49].

*(iii) Aspergillus*. Cavitations secondary to tuberculosis and other chronic infections can provide a nidus for fungus balls, visible as intracavitary soft tissue lesions with an air meniscus on CT [6]. Although there is a significant overlap in the appearances of aspergilloma and malignancy, dependent location, adjacent bronchiectasis, and nonenhancement of the mass on contrast favor aspergilloma [50]. Other forms of* Aspergillus* infections include allergic bronchopulmonary aspergillosis (ABPA) in patients with chronic airway disease and invasive and semi-invasive pulmonary aspergillosis seen in immunocompromised patients. ABPA may cause proximal central bronchiectasis, due to bronchial damage. In invasive pulmonary aspergillosis, air crescents on CT result from cavitary necrosis of infected tissue presenting with a characteristic halo sign and indicate immune system recovery [6]. The soft tissue density in invasive aspergillosis represents necrotic tissue and is not freely movable as in the case of an aspergilloma. Chronic necrotizing pulmonary aspergillosis is another form that is associated with cavitary infiltrates that occur in patients with chronic lung disease and immunosuppressive conditions such as alcoholism and diabetes [30].

#### 2.2.2. Caseous

*(i) Tuberculosis*. Tuberculomas are granulomas usually less than 3 cm in diameter, frequently seen in an upper lobe [40]. These lesions are characterized by a lack of temporal progression and evidence of dystrophic calcification. Long-standing tuberculomas undergo caseous necrosis resulting in expulsion of necrotic debris into the bronchial airways. Caseous necrosis has also been described in other fungal infections like cryptococcosis, coccidioidomycosis, and histoplasmosis [51]. The walls of an untreated tuberculous cavity are usually thick and irregular [5]. Calcified hilar lymphadenopathy supports the diagnosis. Nontuberculous mycobacterial (NTM) infections are also known to cause cysts which are more thin-walled and surrounded by less dense consolidation when compared with tuberculous lesions [5]. The presence of cavitation in association with right middle lobe and lingular bronchiectasis and nodules often suggests nontuberculous mycobacterial disease. Cystic lesions and bronchiectasis are more characteristic CT findings in NTM infections and help in differentiation [52].

A mimicker of tuberculomas is actinomycosis which typically manifests as an area of persistent consolidation or a mass, either of which may contain cavitation [41]. Chest wall invasion may occur. Bronchogenic carcinoma must also be considered as it may present as a cavitary lesion (see below).

#### 2.2.3. Ischemic

*(i) Autoimmune/Rheumatologic Diseases*. Among the autoimmune disorders, Wegener's granulomatosis with angiitis and rheumatoid disease is most commonly associated with cavitations [5, 30]. Lung involvement in rheumatoid arthritis is not uncommon and may present as necrobiotic nodules which are characteristically multiple, peripheral, and thick-walled, resolving with treatment [53] (Figure 13). Wegener's granulomatosis with angiitis is a clinicopathological entity characterized by necrotizing granulomas involving the upper and lower respiratory tract causing cavitations in up to half the cases of lung involvement [54]. Cavitations occasionally occur within nodules distributed along the bronchovascular bundles, interlobular septa, major fissures, and subpleural regions in pulmonary sarcoidosis [34]. Cavities are less frequently encountered in other autoimmune diseases and are more likely to be infectious in origin from immunosuppressive use [44].

*(ii) Amyloidosis*. AL is generally found in primary amyloidosis in association with monoclonal plasma cell proliferation such as multiple myeloma, whereas protein AA is the major component of secondary amyloidosis, a feature of chronic inflammatory disorders [33]. Amyloidosis can present in the lung in three forms: nodular, diffuse parenchymal, and tracheobronchial. The formation of cysts is a late and relatively rare manifestation and they occur most commonly in localized amyloidosis in association with Sjögren's syndrome, resulting from peribronchiolar deposition of amyloid [11]. However, cysts in Sjögren's syndrome may also present secondary to LIP (see section on LIP) [55]. It must be remembered that Sjögren's syndrome can present with a wide range of interstitial patterns including nonspecific interstitial pneumonia, lymphocytic interstitial pneumonia, usual interstitial pneumonia, and organizing pneumonia. Pulmonary amyloidosis accompanying Sjögren's syndrome and lymphoproliferative disease generally manifests as multiple, large, thin-walled cysts and nodules in areas of lymphocyte predominance which include the interlobular septae, bronchovascular interstitium, and subpleural areas; honeycombing and traction bronchiectasis may also be seen [11, 55]. Such cases may be differentiated from bronchiectasis in which the cysts have thicker walls and are more centrally located [33]. Calcified nodules on CT are a fairly characteristic finding in amyloidosis.

#### 2.2.4. Intratumoral

*(i) Neoplasms*. A study by Woodring et al. [12] showed that it is possible to make a specific diagnosis of benignancy or malignancy based on the measurement of the thickest part of the cavity wall, with lesions with less than 4 mm of wall thickness considered as benign and greater than 15 mm of wall thickness considered as malignant. Chronic cavities (greater than 1month in duration) are more likely to be malignant, congenital, or a result of chronic inflammatory disorders; acute cavities (less than 1 month from onset) are often infectious, inflammatory, septic embolic, or traumatic in origin [3].

Cavitary neoplasms are formed by central necrosis with expulsion of the necrotic tumor debris into the communicating airways. Other mechanisms for cyst formation in both primary and metastatic lung tumors include check valve effects (formed by neoplastic stenosis) and destruction of the alveolar sacs. Uniformly distended progressively enlarging cysts on serial CT scans suggest check valve effects from micrometastastic tumor lesions. Also, tumors in the lung can masquerade as abscesses or other forms of benign cysts [18]. A tumor may very rarely present as benign looking thin-walled cyst [56]. Hence, early and complete resection of benign lung cysts is advisable [18]. Alternatively, a high degree of suspicion is required and careful detection of the changes in clinical and radiologic findings over time is important to avoid missing the diagnosis [56]. Cavitary tumor lesions in the lung are usually from squamous cell carcinomas [5] (Figure 9). Lung adenocarcinoma can rarely present with cystic lesions and should be suspected in patients with mediastinal masses and diffuse cystic lesions in the lung [53] (Figure 10). Endobronchial neoplasms may also cause a solitary cyst via a check valve mechanism. Cysts may also develop as a postchemotherapy effect due to cavitary necrosis of solid tumor masses [57].

Cystic metastases have been described in endometrial, pancreatic, and colon cancers. Cystic lung metastases occur more frequently in tumors of epithelial origin and less frequently in tumors of mesenchymal and hematopoietic origin. Nevertheless, cystic lesions have been described in metastatic osteosarcoma [58], leiomyosarcoma [59], synovial sarcoma [59], and epithelioid sarcoma [60], among others.

*(ii) Airway Papillomatosis*. Squamous cell papillomata are benign proliferation of squamous mucosa. Papillomata are the most common benign tumors in the laryngotracheal region [61]. Lesions are often limited to the nasopharynx but can descend to involve the tracheobronchial tree and lung by microaspiration or a multicentric origin, causing small solid or cavitary lesions (Figure 11). Cysts may result either from obstructive emphysema or from cavitatory necrosis of the papillomatous lesions [5].

### 2.3. Alveolar Rupture and Air Space Confluence

**Figure 7:**
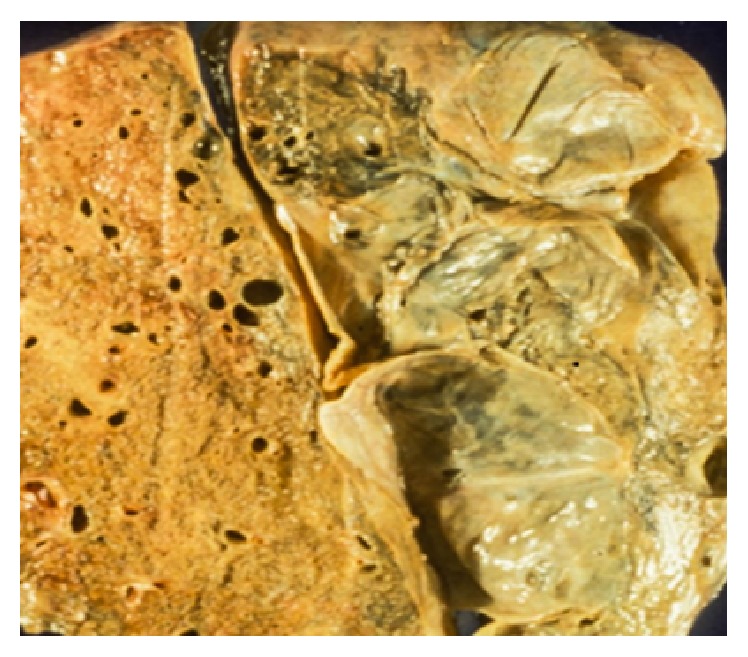
Gross specimen showing lung tissue with scattered thin-walled bullae. Large bullous cavity resulting from expansion of one of the bullous air spaces.

*(i) Emphysema*. Emphysema is characterized by permanently enlarged air spaces distal to the terminal bronchiole with destruction of the alveolar walls [9]. Emphysema may be classified into various types, based on the pattern of lung parenchyma destruction with respect to the secondary pulmonary lobule: proximal acinus in centriacinar (centrilobular) emphysema and distal in paraseptal and whole acinus in panacinar (panlobular) emphysema. Centrilobular type is more often found in upper lung zones (i.e., the posterior and apical segments of the upper lobes or the superior segments of the lower lobes) and is commonly associated with cigarette smoking [62]. The emphysematous centriacinar air spaces over time may conflate into large air spaces with bizarre shapes. Typical findings by CT include irregular, small, round, or confluent areas of low attenuation interspersed within normal lung, often in proximity to the center of a secondary pulmonary artery. The cysts in the centrilobular form of emphysema have central nodularity due to the presence of intralobular pulmonary artery allowing differentiation from the cysts seen in PLCH and LAM [6]. Centriacinar emphysema, unlike panacinar emphysema, is recognizable by its easily discernible contrast from the surrounding normal lung [63]. Other differentiating radiologic features from panlobular emphysema include upper lobe predominance, greater degree of lung inflation, and less frequent bullous formation [64, 65]. Bullous emphysema can occur either in centrilobular or in panlobular emphysema and does not refer to any specific pathological entity. It is simply a more advanced form of emphysema characterized by the presence of blebs/bullae conflated by ruptured pulmonary alveoli. These giant blebs may grow to achieve significantly large volumes occupying an entire lobe with the potential to rupture and complicate as pneumothorax [10, 66] (Figure 7). Bullous emphysema is the most common attributable cause of lung cyst formation [5].

*(ii) Syndromes*

**Figure 8:**
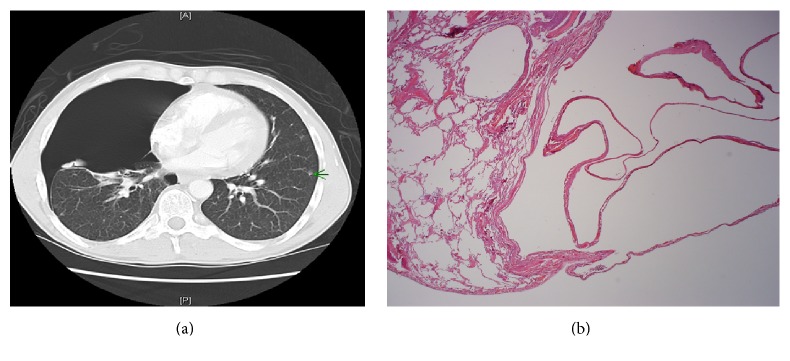
(a) HRCT of the lung showing pneumothorax on the right. Pneumothoraces tend to arise from the rupture of peripherally located cysts as pointed out on the left of the image (green arrow). (b) A low power view (×40) showing the ruptured cyst lined by a thin-walled alveolar epithelium.

**Figure 9:**
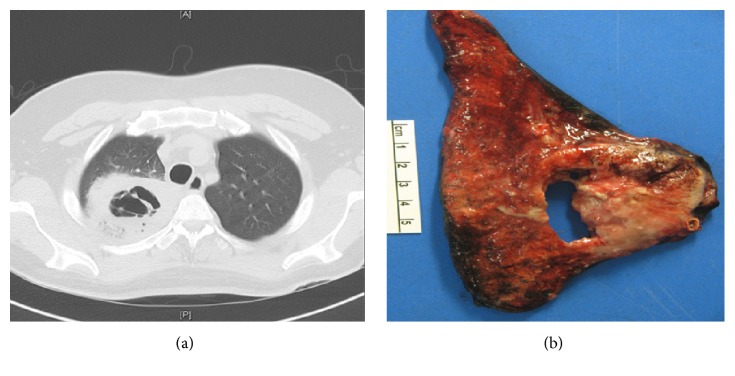
Squamous cell carcinoma. (a) HRCT of the right lung demonstrating a large thick-walled solitary cavitation with internal septations. (b) Gross specimen demonstrating a large area of cavitation with thick surrounding walls from malignant infiltration.

**Figure 10:**
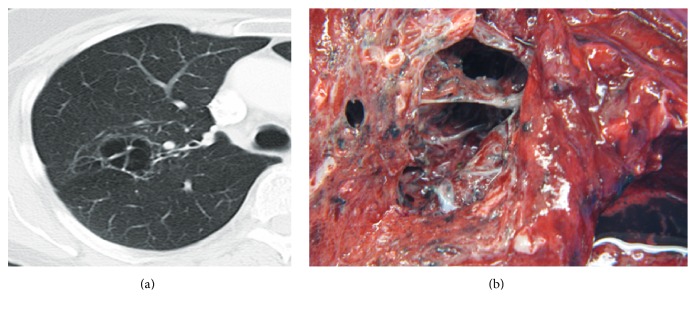
Adenocarcinoma with cystic change. (a) Gross surgical specimen of resected lung tissue showing the cystic mass, with thin-walled septations. (b) High-resolution CT scan of the right lung demonstrates a large, thick-walled cavitary mass in the upper lobe. Multiple thin septations are present within the mass.

**Figure 11:**
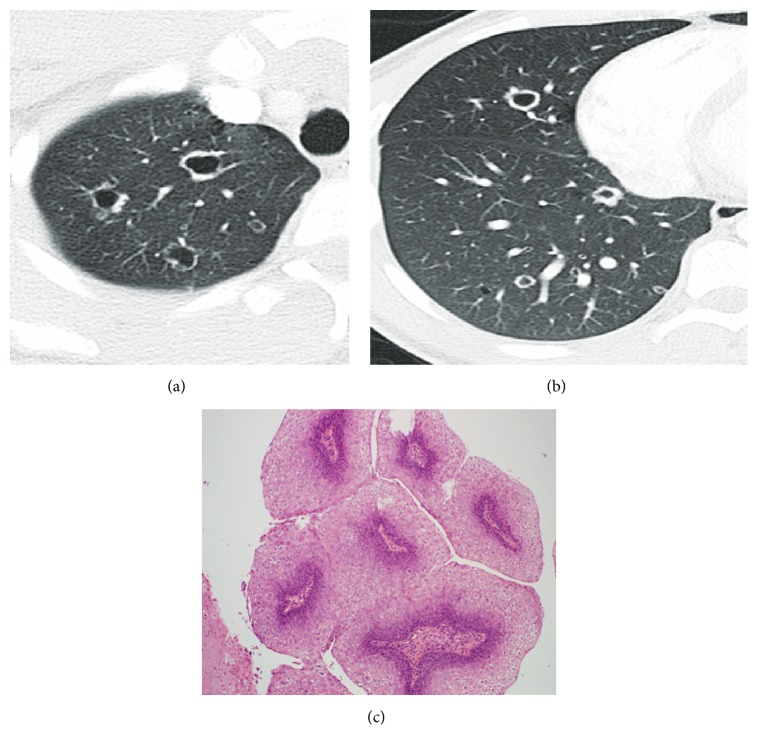
Airway papillomatosis. (a and b) HRCT of the right lung at descending levels reveals multiple pulmonary nodules, many of which have undergone cavitation to form irregular thin-walled cysts. (c) High power view showing papillomata with central fibrovascular core surrounded by benign squamous epithelium with evidence of hyperkeratosis and parakeratosis (magnification ×100).

**Figure 12:**
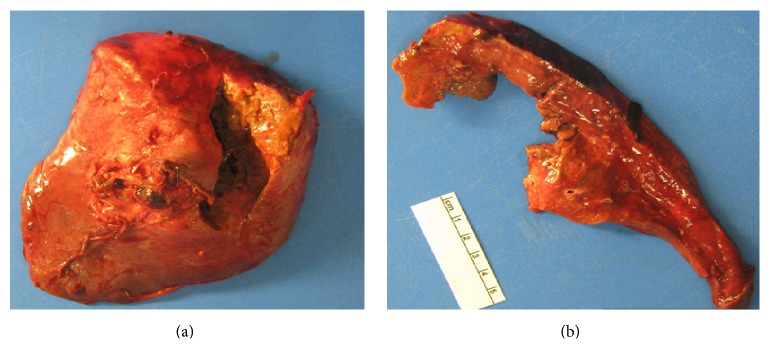
(a) Gross surgical specimen of lung demonstrating a necrotic fungal cavity; (b) cut section of the lung showing an irregular contoured necrotic wall.

**Figure 13:**
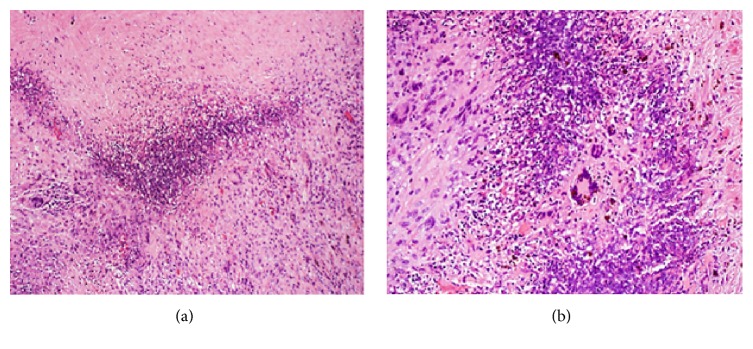
Left: (a) high power view showing coagulative necrosis (upper center) surrounded by multinucleated giant cells in a poorly formed granuloma surrounded by palisading histiocytes (magnification ×100). Right: (b) a higher power view demonstrating necrobiotic granuloma comprising multinucleated giant cells (center of the image), surrounded by palisading histiocytes (magnification ×200).

*Birt-Hogg-Dubé (BHD) Syndrome*. Birt-Hogg-Dubé (BHD) syndrome is an autosomal dominant condition characterized by cutaneous papular skin lesions, multiple lung cysts that may cause pneumothorax, and an increased risk of renal tumors [67]. The syndrome is caused by a deletion of the FLCN gene on chromosome 17 which encodes the protein folliculin. The lung cysts are elongated and oval, closely associated with the peripheral interlobular septum, visceral pleura, or septal-pleural junctional region; the rupture of these cysts may result in pneumothoraces (Figure 8). These cysts are lined by a layer of alveolar epithelium. Several mechanisms have been proposed for the mechanism of formation of the lung cysts in BHD including inflammation and growth failure (secondary to haploinsufficiency of folliculin leading to deranged alveolar development) [68]. Both BHD and lymphangioleiomyomatosis may be seen in middle-aged women. However, unlike the cysts of LAM, those seen in BHD are larger and nonprogressive and are predominant in the lower and medial zones of the lung [68].

*Ehlers-Danlos Syndrome (EDS)*. Ehlers-Danlos syndrome (EDS) is an inherited disorder of connective tissue with multiple thoracic manifestations. One of the thoracic manifestations of EDS is the presence of parenchymal cysts. The cysts are due to formation of hematomas with subsequent cavitation which may result in hemoptysis following communication with the tracheobronchial tree [69]. Repair by osseous metaplasia results in the formation of fibroosseous nodules, a rather unusual thoracic manifestation [69].

*(iii) Traumatic*. Posttraumatic pneumatoceles (traumatic pulmonary pseudocysts) following blunt thoracic trauma are not frequently observed [37, 47]. Pulmonary contusion is the usual pattern of lung injury following blunt chest trauma. The mechanism of cyst formation appears to be due to inciting trauma with subsequent elastic recoil of the normal surrounding parenchyma creating clefts between the normal and injured parenchymata [37]. These clefts may be alveolar or interstitial. Pneumatoceles may also form secondary to increased intrapulmonary pressure due to positive pressure ventilation. These are typically transient and resolve with time.

### 2.4. Cystic Expansion of Structures with Lung Displacement

#### 2.4.1. Infectious

*Parasitic Cysts*

*Hydatid Cyst*. Hydatid cysts primarily involve the liver. In the case of pulmonary involvement, a simple hydatid cyst is most common in the right lower lobe [42], though it may arise anywhere including the upper lobes. Cysts in the lung are commonly multiple with wall thickness from 0.2 to 1 cm. CT and radiography are the diagnostic methods of choice, considering high false positive results with serodiagnosis [42]. Bronchiolar erosion into the pericyst may introduce air between the pericyst and the laminated membrane. These air collections appear as radiolucent crescents in the upper part of the cyst and are known as the crescent sign. Demonstration of air bubbles (formed by air dissecting between the pericyst and the parasitic membrane) with ring enhancement is a strong indicator of infected hydatid cysts [42].

*Paragonimiasis*. Paragonimiasis is an infection caused by lung flukes of the genus* Paragonimus*. In Asia,* P. westermani* infections are relatively common where diets include raw or salted crustaceans. Mammals acquire the infection when they ingest raw or undercooked crustaceans [70]. Features seen on CT include linear opacities (due to worm migration tracks or focal atelectasis due to obstruction of the airway lumina); focal air space consolidation (due to hemorrhagic pneumonia, as the parasite travels through the parenchyma); and cysts due to the infarction by blocking of the arteriole or vein by the expansion of the parasite in the lung tissue [43]. Clinicians should consider the diagnosis of paragonimiasis in all patients with cough, fever, and pleural effusion with peripheral eosinophilia [70].

#### 2.4.2. Congenital/Hamartomatous

Congenital cystic lesions typically present during childhood but may rarely manifest in adults. These lesions must be distinguished from other acquired adult cystic diseases. A brief discussion on the more common congenital cystic diseases is included.

*(i) Congenital Pulmonary Airway Malformation (CPAM), Formally Congenital Cystic Adenomatoid Malformation (CCAM)*. CPAM is rare in adults and can be a frequently underdiagnosed entity. Bronchoalveolar dysgenesis during the first trimester results in adenomatoid hamartomatous proliferation of terminal bronchiolar units (hence its former name). Histologically, the CPAM lesions are characterized by bronchiolar proliferation and columnar or cuboidal epithelium lined micro- and macrocysts [71]. CPAM was originally classified into 3 types, by Stocker, and was later revised to include Type 0 and Type 4 [72, 73]. Type 1 is the most common form with large cysts ranging from 2 to 10 cm in diameter. Type 1 CPAM is a known precursor for mucinous adenocarcinoma [74]. Type 4 CPAM can present as a solitary cyst typically involving a single lobe [73]. These lesions, unlike bronchogenic cysts, have a connection with the central airways. Lack of reliable differentiating characteristics, due to shared pathogenesis, sometimes necessitates histologic exam in order to rule out the latter. Additionally, histological diagnosis may be difficult to make due to architectural distortions from prior infections [71].

*(ii) Bronchogenic Cysts*. Bronchogenic cysts (BC) represent congenital abnormalities resulting from abnormal budding of the developing tracheobronchial tree. While they typically appear as intraparenchymal masses, they may also manifest as cystic cavities, usually within the lower lobes [30]. As alluded to, they do not communicate with the tracheobronchial tree. However, supervening infection may establish a communication with airways allowing the cysts to enlarge rapidly [3].

**Figure 14:**
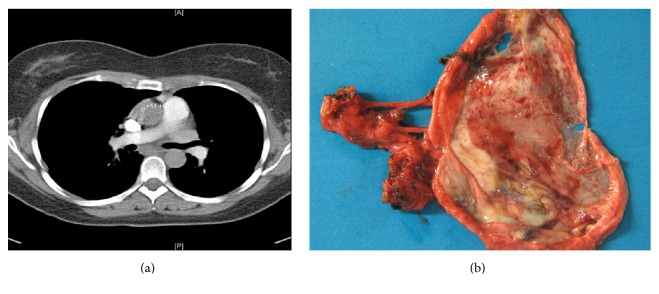
(a) Bronchogenic cyst. High-resolution CT scan reveals a 5.0 × 7.0 cm, well-circumscribed, thin-walled cystic mass of fluid density (+51 Hounsfield units) arising within the right lung and abutting the posterior aspect of the heart. There is no communication with the bronchus. (b) Gross specimen of a bronchogenic cyst bisected to reveal a smooth inner surface with punctate hemorrhagic foci and mucoid material and interrupted by trabeculations.

The high attenuation of bronchogenic cysts on CT scans is caused by hemorrhage, mucoid proteinaceous fluid, or calcium oxalate [73, 75] (see Figure 14). These cysts may be intrapulmonary or mediastinal in location [75]. These cysts are frequently superinfected further complicating the clinical picture. Sometimes, definitive diagnosis is only possible by coupled histopathological examination. Along these lines, the most reliable criterion for the diagnosis of intrapulmonary BC is considered as the presence of cartilage in the cyst wall.

*(iii) Sequestration*. Bronchopulmonary sequestration refers to the isolation of a portion of the lung with development under its own arterial supply. Sequestration is a rare phenomenon in adults [76, 77]. Two variants exist based on whether visceral pleura encompasses the isolated segment along with (intralobar sequestration) or distinctly from (extralobar sequestration) the main [30, 73]; 75% of sequestrations are intralobar with the majority of them occurring in the basilar segment of the left and right lower lobes [78]. Intralobar sequestrations are complicated by recurrent infections and bronchial obstruction. As in intralobar sequestration, extralobar sequestration occurs most often on the left, is often peripheral, and presents as a wedge-shaped density associated with the left diaphragm in almost 90% of cases [3]. A definitive diagnosis can be made by demonstrating the aberrant systemic arterial blood supply as a linearly enhancing structure adjacent to the aorta on contrast CT [79]. Diagnosis requires maintaining a high degree of clinical suspicion in young and middle-aged patients with left lower lobe cystic lesions.

## 3. Conclusions

In this review, we provided an overview of the diseases most commonly associated with cystic lung lesions in adults, by illustration and description of pathologic and radiologic features of each entity. We also classified pulmonary cystic disease with an emphasis on pathophysiology behind cyst formation in an attempt to explain the characteristics of similar cystic appearances seen in various disease entities. It is possible to make a fairly specific diagnosis during early stages of disease evolution by its relatively distinct radiological presentation which is very well appreciated on high-resolution CT scans. Distinct pathologic presentations caused by a variety of insults to the lung, over time, progress to converge into relatively nonspecific chronic injury patterns characterized by cysts among other features. Although specificities of HRCTs are much improved with resultant decrease for the need of surgical biopsies, microscopic analysis may provide the only method of diagnosis during the later stages of lung injury where gross pathologic and radiologic manifestations tend to be relatively nonspecific. Some of these diseases may manifest rarely with atypical and unusual presentations, requiring a multidisciplinary approach to arrive at an accurate diagnosis.

## Figures and Tables

**Table 1:** Cysts and cyst-like lesions based on the mechanism of cyst formation^∧^.

Mechanism of cyst formation	Principal pathophysiology	Associated diseases	Other features
Cystic dilatation of lung structures	Ball valve effect due to proximal obstruction by peribronchiolar infiltration	*LAM* [11] *Lymphoid Interstitial pneumonia* [17, 19, 20] * PLCH* [29]^*∗*^ *RB-ILD* [7, 16] *DIP *[25] *P. jirovecii* [26] Primary neoplasms Endobronchial neoplasms Metastasis or micrometastases Lymphoma [25]^*∗∗∗∗*^	Generally thin-walled (<4 mm)^3^, uniform in size, and rounded in shape. May change size depending on phase of respiration due to communication with airways [5]; infiltration/nodules may be observed either radiologically or histopathologically proximal to cysts. May be diffuse (as in LAM, PLCH, and LIP) [6, 17] or focal (as in solitary neoplasms) [18, 56].

Cystic dilatation of lung structures	Traction bronchiolectasis or alveolar ectasia due to retraction from surrounding interstitial fibrosis	*Honeycombing UIP/IPF* [7] *Collagen vascular disorders*, *asbestosis*, *hypersensitive pneumonitis*, and *drug induced fibrosis* [56] *PLCH* [29]^*∗*^ *Viral bronchiolitis* [5] *Follicular bronchiolitis* [17] Lymphoma [25]^*∗∗∗∗*^	Air spaces are clustered to resemble a honeycomb. Cysts are associated with other features indicating fibrosis including architectural distortion, interstitial thickening, and traction bronchiectasis. Granuloma induced fibrosis in PLCH results in irregular shaped cysts when compared to the more uniform shaped cysts as in LAM.

Cystic dilatation of lung structures	Cystic bronchiectatic air spaces formed due to fibrosing dilatation of the bronchi as a result of suppurative and/or necrotizing inflammation	*Chronic infections*, *immunodeficiency disorders*, *genetic syndromes*, and* cystic fibrosis *[5, 31]	Air spaces can be confused for cysts when viewed “en face.” The bronchi have thick walls and do not taper when followed to the periphery. Cysts are more central than bullae and may contain fluid and change size with phase of respiration due to communication with airways [5].

Parenchymal necrosis	Suppurative necrosis	*Abscesses (bacterial and amoebic) *[45] *Fungal infections*, *nontuberculous mycobacterial infections* [52] *Postinfectious pneumatoceles* [46]	Thick-walled (>4 mm) with irregular margins seen as low areas of attenuation within an area of pulmonary consolidation or nodule on CT^3^. Air fluid levels present due to superimposed infection. May be thin-walled in resolved consolidations as seen in pneumatoceles [46].

Parenchymal necrosis	Caseous necrosis	*Tuberculosis*, *cryptococcosis*, *coccidioidomycosis*, and *histoplasmosis* [51]	Thick-walled cavitations with irregular margins predominantly in upper lobes; NTM cavitations more thin-walled and commonly associated with bronchiectasis [40, 52, 80].

Parenchymal necrosis	Ischemic necrosis	*Pulmonary infarcts* *Vasculitides *[54] *Amyloidosis *[33]^*∗∗*^ *Rheumatic disorders *[53] PLCH^*∗*^	Multiple or solitary thick-walled cavitations depending on the extent of involvement.

Parenchymal necrosis	Desquamation of tumor tissue with subsequent liquefaction	*Malignancies* *Primary*^*∗∗∗*^ *Squamous cell carcinoma *[56]*, adenocarcinoma* [18, 81] *Secondary pulmonary lymphoma*, *AIDS lymphoma *[17] *Metastasis Mesenchymal sarcomas *[58, 59], *epithelial carcinomas *[60, 82], and *airway papillomatosis* [61]	Cavities are typically thick-walled and are usually found in an area of mass or nodule, multifocal or solitary. Very rarely thin-walled indicating formation by other mechanisms and can present as a diagnostic pitfall [83].

Alveolar rupture and/or confluence of air spaces	Alveolar dissolution from ischemia or alveolar rupture and further conflation resulting in large air spaces	*Emphysematous bullae COPD*, *connective tissue syndromes (e.g*., *EDS *[69])Later stages of MCLD including PLCH,* amyloidosis* *BHD syndrome *[68]	Blebs and bullae have imperceptible walls (<1 mm). Tend to be peripheral and do not communicate with airways [5]. Bizarre large cysts are seen in later stages due to confluence of cystic air spaces [29].

Cystic expansion with lung displacement	Parasitic cysts	*Parasitic cysts Echinococcosis* [42] *Paragonimiasis* [43]	See description in Sections 2 and 3.

Cystic expansion with lung displacement	Congenital cyst formations manifesting in adults	*CPAM* *Bronchogenic cyst *[75] *Sequestration* [73]	Cysts may present very similar to acquired cystic lesions.

Miscellaneous	Iatrogenic herniation of the bowel into the thorax	*Lucite plombage* [5] *Traumatic diaphragmatic rupture *[84] *Bochdalek hernia* [85]	Apical distribution, in resistant tuberculosis. Herniation of abdominal structures into the thorax, with a cystic appearance on CT.

^∧^Diseases whose cystic formation is predominated by the particular mechanism are written in italics.   ^*∗*^Different mechanisms involved in cyst formation including bronchiolectasis and cavitary necrosis of nodules in early stages with confluence of air spaces in end stages of disease.  ^*∗∗*^Various mechanisms proposed for cysts in amyloidosis including air trapping, ischemia by amyloid deposits on vascular walls, and bronchiolectasis.  ^*∗∗∗*^Majority of the solitary/multifocal neoplastic cavitations are due to squamous cell neoplasms and rarely due to adenocarcinomas.  ^*∗∗∗∗*^Rarely reported in adult lymphomas from check valve and traction fibrosis.

MCLD: multiple cystic lung disorder; IPF: idiopathic pulmonary fibrosis; LIP: lymphocytic interstitial pneumonia; LAM: lymphangioleiomyomatosis; PLCH: pulmonary Langerhans cell histiocytosis; HRCT: high-resolution CT scan; DIP: desquamative interstitial pneumonia; RB-ILD: respiratory bronchiolitis interstitial lung disorder; OP: organizing pneumonia.
